# Morphological priming development in Brazilian Portuguese-speaking children

**DOI:** 10.1186/s41155-017-0058-8

**Published:** 2017-02-20

**Authors:** Bruno Stefani Ferreira de Oliveira, Francis Ricardo dos Reis Justi

**Affiliations:** Department of Psychology, Federal University of Juiz de Fora, Juiz de Fora, MG 36036-330 Brazil

**Keywords:** Morphological processing, Morphological priming, Morphological awareness, Reading, Literacy

## Abstract

Many studies have shown that, while reading, the brain works a sort of decomposing process of words, reducing them to its morphemes. Moreover, many studies have shown that morphological awareness, which is the ability to reflect upon the structure of words, may contribute to reading in Brazilian Portuguese. The present study investigated morphological priming in children and evaluated the correlation between morphological priming and morphological awareness scores. One hundred and forty-one children took part in this research: 35 second graders, 33 third graders, 33 fourth graders, and 40 fifth graders. They performed a lexical decision task (LDT) and morphological awareness task. Results indicate that as early as the second grade, children show some degree of morphological priming effects. In addition, children from the fifth grade presented morphological priming effects similar to those of a skilled reader. No correlations between morphological awareness scores and morphological priming effects were found.

**Electronic supplementary material:**

The online version of this article (doi:10.1186/s41155-017-0058-8) contains supplementary material, which is available to authorized users.

## Background

As the act of driving is to an experienced driver, the skilled reader is able to quickly decode a series of letters and extract the representation of its meaning without great efforts. This is such an automated behavior that people generally do not realize how complex the reading process can be. Over the past 40 years, many researchers have been investigating whether the brain processes a word as a whole or decomposes it into its morphemic components and then regains its meaning (Amenta et al. 2015; Domínguez et al. 2000; Miller et al. 2016).

According to Domínguez et al. (2000), there are three theoretical models that propose different solutions for the role of morphological structure in skilled reading. The first model is called full listing, and it defends that words are available for recognition in the mental lexicon in its full form, without any morphological decomposition (Domínguez et al. 2000). In this case, there would be direct access from inputs which are associated to orthographic and phonological forms stored in the lexicon. A second type of model is called full parsing. In this model, words are processed through the decomposition to its morphemic units and only after this, does the access to meaning happen (Taft and Forster 1975). For instance, the recognition of the word “helper” initially happens through the isolation of the morphemes “help” and “er”. Only then is the meaning reached: a person who makes it easier for someone to do something. This sub-lexical process would help the identification of new words through its morphemes and would also save resources in the lexicon once there would be no need to store all words in their full forms. Finally, another model to identify words, as stated by Dominguez et al. is the dual or mixed model, which is a combination of full listing and full parsing. In this model, there are two parallel routes during the identification of words. Therefore, the speed to access the meaning is related to the type of word presented. The most frequent words would generally be processed faster by direct access, since the decomposition would delay their identification. On the other hand, unknown words and words that are rarely seen would be identified by morphological decomposition, which could be a key way to comprehend the meaning of a new word.

An important task used to investigate lexical access during reading is the lexical decision task (LDT). In this task, the participant must decide in the quickest and most precise possible way if the stimulus is a word or a pseudoword. A pseudoword is a sequence of letters that respects orthographic and phonotactic rules of the target language but does not have meaning, e.g., “vonk” is a pseudoword in English. According to Domínguez et al. (2000) and Amenta and Crepaldi (2012), LDT studies show that pseudowords consisting of both a real root and a real affix, e.g., “dischant,” are rejected more slowly than those without any morphological structure, e.g., “donrapel.” This has been taken as evidence for a pre-lexical processing of words’ morphology because if morphology is only processed after the stimulus is recognized, then the reaction time to discard a pseudoword should not be affected by its morphology.

In psychological literature, priming refers to the effect that a prior stimulus (prime) has on the processing of the target stimulus (Kantowitz et al. 2006). In general, the priming tasks manipulate different time intervals between the prime presentation and the beginning of the target. This interval is called SOA (stimulus onset asynchrony), and it might be so short that the individual does not consciously perceive the stimulus (usually at an average of 50 ms) or long enough for the prime to be detectable (usually above 200 ms). Research in different languages (e.g., Amenta et al. 2015; Grainger et al. 1991; Drews and Zwitserlood 1995; Miller, Liran-Razan and Vaknin, 2016) points to evidence of morphological priming effects. Pairs of morphologically related words presented shorter reaction times in comparison to pairs of unrelated words or pairs that only had orthographic similarities or only semantic similarities. In reviewing those studies, Amenta and Crepaldi (2012) argue that questioning whether or not morphological priming in skilled readers happens does not make sense anymore. The evidence of its existence in certain situations is uncontroversial; the remaining question is which variables affect morphological priming.

One of the variables that might modulate morphological priming is the SOA. Rastle and Davis (2008) revised research conducted in different languages and concluded that the time to recognize a word preceded by a pseudo-derived prime (e.g., return, research) compared to the time to recognize a word preceded by a morphological prime (e.g., study, student) is not different for short SOAs. Nevertheless, in studies where the SOA was longer, words preceded by morphological primes were recognized faster than words preceded by a pseudo-derived prime. In order to explain these results, Rastle and Davis (2008) proposed two distinct moments in the visual processing of words: a morpho-orthographic moment, which would happen during the initial phase of perception (usually measured in studies with short SOAs), and a morpho-semantic moment, which would happen after that (usually measured in studies with longer SOAs).

The morphological priming effect has also been investigated in young readers as a way to identify its onset. For instance, Casalis et al. (2009) used a priming paradigm with the LDT to investigate the SOA and morphological prime effects. Fifty-three French fourth-grade children were part of that study. They were divided into two groups, according to SOA duration: 75 or 250 ms. The prime words were selected under three conditions according to the relation with the target: morphological, orthographic, and unrelated. All words were regular regarding grapheme-phoneme mapping. There was a priming effect of similar size for both morphologically and orthographic-related pairs in the 75 ms SOA. However, for the 250 ms SOA, the prime effect was only observed for morphologically related pairs.

Quémart et al. (2011) performed three experiments with the priming paradigm in LDT to investigate the development of morphological processing in French. Children from third, fifth, and seventh grades and adults took part in this study. The prime manipulation consisted of four different types of relation between the prime and the target word: morphological, pseudo-derivative, orthographic, and semantic. The SOA manipulation was between subjects and had three levels: 60, 250, and 800 ms. For the two shortest SOAs, only morphological and pseudo-derivative primes had effects, that is, target words preceded by these primes were recognized faster. For the longest SOA (800 ms), participants were faster for words preceded by morphological, semantic, and orthographic primes but not for pseudo-derivative primes. There was no difference in the priming patterns between the grades. These results from Quémart et al. (2011) study suggest that morphological processing occurs from third grade and that developing readers present both a morpho-orthographic priming effect (when the SOA is short) and a morpho-semantic priming effect (when the SOA is longer).

It is important to consider that, although there is evidence of morphological priming in young readers (Casalis et al. 2009; Quémart et al. 2011), it is still not clear when the effect starts and if it occurs in more transparent languages. For example, the youngest group of children from Quémart et al. (2011) study had a mean age of 8 years and 10 months. Thus, one may argue that those kids have been exposed to systematic reading teaching for at least 2 years. Furthermore, the participants from Casalis et al. (2009) and Quémart et al. (2011) studies were French-speaking children and adults. Therefore, it is also important to investigate if morphological priming occurs (and if so, when it occurs) in Brazilian Portuguese, considering the grapheme-phoneme mapping, is a more transparent language than French (Seymour 2005). This is relevant because, as previously argued by Chomsky and Halle (1968), in less transparent languages (such as English and French), morphology would be important for reading irregular words (that is, words that do not follow the languages’ grapheme-phoneme conversion rules). Since in Brazilian Portuguese there are few irregular words, it is possible that morphological processing does not play an important role in reading this language. Therefore, the central goal of the current study is to investigate the development of morphological priming in the reading of Brazilian Portuguese children from second to fifth grade. Since the second grade is officially the period when the systematic teaching of reading starts in Brazil, the present study would be able to clarify when priming processing has its onset.

Currently, there have only been two studies about morphological priming performed in Brazilian Portuguese speakers, which are Garcia et al. (2012) and Moraes, Angela Mafra De. (2015) In Garcia et al. study, the researchers performed a LDT experiment using the priming paradigm with a short SOA (38 ms). The priming manipulation had four conditions: morphological relation (FILA—fileira), strictly semantic relation (ORDEM—fileira), strictly phonological relation (FILÉ—fileira), and neutral relation (MATO—fileira). Considering the pseudowords, the manipulation had two conditions: with morphemes (ZALA—zaleira) and without morphemes (FUBO—fubila). The participants were college level students. When compared to all other conditions, faster reaction times were observed for the morphological prime condition. The other conditions did not differ from the neutral condition, which suggests that the morphological relation was the only one to facilitate the response to the target. In addition, the pseudowords with morphemes took longer to be rejected. Those findings show that in Portuguese, the presentation of a prime stimulus that is morphologically related to the target word significantly contributes to the recognition of this word. However, as the participants of the study were undergrad students, it does not clarify when the morphological prime effect has its onset in Brazilian Portuguese.

The Moraes, Angela Mafra De. (2015) study was conducted with Brazilian adolescents, and its main goal was to compare dyslexics and controls in LDT performance. The LDT was conducted using a priming paradigm in which prime-target pairs shared four relationships: morphological, pseudoderivation, orthographic control, and semantic control. There was an overall difference in reaction time between the two groups with dyslexics being slower, but there was no difference between priming conditions. However, it is important to notice that the study sample was very small consisting only of 12 participants: six dyslexics and six controls. Therefore, it is not clear if de Moraes results are representative and, as the participants of the study were adolescents, it does not clarify when the morphological prime effect has its onset in Brazilian Portuguese.

Although there are no studies regarding morphological priming in Brazilian Portuguese-speaking children when learning to read, there are many studies showing that morphological awareness may contribute to the reading process in this language (Mota, Anibal et al. 2008; Mota and Silva 2007; Mota et al. 2012; however, see Justi and Roazzi 2012 for a failure to replicate such findings). Morphological awareness is the ability to manipulate and reflect upon the morphological structures of the language (Carlisle 1995). Many studies conducted in different languages have shown a significant relation between scores in morphological awareness tasks and scores in reading tasks (Carlisle 2004; Carlisle and Fleming 2003; McBride-Chang et al. 2005; Kirby et al. 2012). To our knowledge, however, the relation between morphological awareness and morphological priming has never been investigated. Nor is there an explicit theoretical model about this relationship. It is surprising because it is possible to wonder that a good morphological awareness would facilitate the abstraction of morphological units during reading resulting in earlier morphological priming effects. Therefore, an additional goal of the present study is to explore if there is any correlation between morphological priming and morphological awareness in Brazilian Portuguese.

## Methods

### Participants

One hundred and forty-one children participated in the present research; 35-s graders (mean age = 8.0 years; SD = 0.32), 33 third graders (mean age = 8.9 years; SD = 0.41), 33 fourth graders (mean age = 9.8 years; SD = 0.41), and 40 fifth graders (mean age = 11.0 years; SD = 0.39). They were enrolled in two different private schools in a median size Brazilian city. This study is part of the first author’s master’s dissertation and was approved by the Ethics Research Committee of a Brazilian Federal University.

### Materials

*Lexical Decision Task (LDT) stimuli*. The experimental stimuli consisted of 60 words that followed a 3 × 2 factorial manipulation of prime and SOA. SOA had two levels: 60 and 250 ms. Prime had three levels: morphological prime (e.g., *fraqueza-FRACO*); orthographic prime (e.g., *franqueza-FRACO*), and non-related prime (e.g., *espelho-FRACO*). All target words are of average frequency according to Pinheiro (1996) norms. The pairs of words with morphological relation had in average the same number of letters shared in comparison to the pairs of words with orthographic relation. For each school grade, a different list of words was created in order to control the frequency of the pairs of prime-target words. Each list also contained 60 pseudowords that were created for the purpose of the lexical decision task and had a similar number of letters compared to the target words. Moreover, the primes of the pseudowords were formed by real words (e.g., *papel*-CADIA); thus, the type of prime would not indicate the type of target. All together, each list had 120 target stimuli (Additional file 1). In addition to those, the experiment also included 12 other pairs of stimuli for the training session. These pairs had similar characteristics to the words and pseudowords used in the experimental session. Finally, the 60 words that correspond to prime and SOA factorial manipulation were subdivided into six lists so that in each list, a word was preceded by a type of prime (morphological, orthographic, or non-related) and a type of SOA (60 or 250 ms). The order of presentation of those lists was counterbalanced across the six experimental conditions. That way, each participant was exposed to only one list by experimental condition. Therefore, during the experimental session, each student was exposed to 60 words and 60 pseudowords.

*Derived Word Analogy Task*—(task by Nunes, Bryant, and Bindman 1997, adapted by Justi and Roazzi 2012). In this task, when given a pair of morphologically related words, e.g., library—librarian, a new word is presented, e.g., music, and the child must answer using analogy which word completes the second pair, in this case, musician. The score for this task is the number of items answered correctly. According to Mota, Gontijo et al. (2008), the Derived word anology task is one of the best tasks to evaluate morphological awareness in Brazil. It is important to note that Justi and Roazzi (2012) reported a Cronbach’s alpha of 0.65 for this task, which is virtually identical to the 0.64 Cronbach’s alpha reported by Mota, Gontijo et al. (2008). In the present study, we also applied a Morpho-Semantic Decision task as recommended by Mota et al. However, this task resulted in a clear ceiling effect. Therefore, in order to save space, we opted to present and discuss only the Derived Word Analogy task.

### Procedure

The tasks were applied on different days in individual sessions. Considering the Lexical Decision Task, the instruments used were two 15.4-in. screen laptop computers and the DMDX software (Forster & Forster, 2003) was used for the presentation of stimuli and data registry (reaction time and error percentage). The participant sat on a chair at approximately a 40-cm distance from the computer, depending on their posture. After finding a comfortable position, the task procedures were explained to them. They were explicitly told to press the green button on the keyboard only upon seeing a known word written in CAPITAL letters. After that, the participant was asked to read the instructions and had the opportunity to ask any questions before the training session or right after it. The goal of the training session was to make the participants familiar to the buttons and the task, and it was repeated if the participant had an error percentage higher than 35%.

The stimuli were presented on a screen configuration of 640 × 480 pixels, font “*fixedsys*,” size 10 in capital letters. The font color was white and the background was blue. The presentation of stimuli complied the following steps: First, a fixation mark (+) was presented for 500 ms, which was followed by a blank screen presented for 500 ms. Then, a mask (######) appeared for 500 ms, being immediately followed by the prime word in lowercase letters that could last for 60 or 250 ms (according to the SOA manipulation). The prime was substituted by the target (a word or a pseudoword) in capital letters. The participants had 4 s to decide if the target was a word by pressing the green button. The stimulus disappeared when the participant pressed the button or after 4 s. The participants were told that if the stimulus was a pseudoword, they just had to wait until the stimulus disappears. The training session followed the same steps; however, there was also a feedback. The total testing time, including training and experimental sessions, was about 12 min per participant.

## Results

Since chance performance in the LDT is about 50%, all participants whose error percentage was over 40% in this task were excluded from data analysis. This resulted in the exclusion of nine participants: 3-s graders, 3 third graders, 1 fourth grader, and 2 fifth graders. As recommended by Perea (1999), all reaction time scores of a participant above or below two standard deviations were restricted to these values. After those procedures, Kolmogorov-Smirnov tests were conducted on LDT’s reaction time data and their distribution was normal. Table 1 presents the descriptive statistics of these variables.

**Table 1:** Descriptive statistics of lexical decision task

		SOA 60 ms			SOA 250 ms		
		NR_P	Ort_P	Morp_P	NR_P	Ort_P	Morp_P
2nd grade	Mean RT	1628.4	1573.4	1530.8	1652	1581.6	1533.0
	SD	440.7	324	304	355.8	285.7	372.3
	*N*	32	32	32	32	32	32
3rd grade	Mean RT	1404.2	1322.7	1334.7	1350.5	1373.1	1264.2
	SD	312.7	308.2	336.1	303.9	312.5	308.5
	*N*	30	30	30	30	30	30
4th grade	Mean RT	1238.7	1244.3	1223.2	1237.7	1252.8	1127.2
	SD	361	373.2	364.1	318.5	347.1	350.4
	*N*	32	32	32	32	32	32
5th grade	Mean RT	1134	1134.9	1071.8	1146.9	1156.5	1070.7
	SD	339.1	319.2	310.8	352.5	330.6	342.1
	*N*	38	38	38	38	38	38

*Abbreviations*: *NR_P* non-related prime, *Ort_P* orthographic prime, *Morp_P* morphological prime, *RT* reaction time, *SD* standard deviation

An ANOVA was used to analyze the second-grader data, and the results show that neither the main effect of SOA nor the interaction SOA × Prime were statistically significant (all *p* values >0.77). There was a significant effect of Prime, *F*(2,62) = 3.69, *p* = 0.031. Planned comparisons were performed in all school grades between the morphological prime and the non-related prime and between the morphological prime and the orthographic prime for each type of SOA. The results indicate that the difference between non-related prime and morphological prime was significant when the SOA was 250 ms: *t*(31) = 2.62, *p* = 0.01. The words preceded by morphological primes were recognized 119 ms faster than those preceded by non-related primes. No further significant results were yielded for the other comparisons (all *p* > 0.12).

For third graders, ANOVA analysis indicates that the main SOA effect and the SOA × Prime interaction were not statistically significant (values of *p* > 0.20). The only statistically significant effect was that of Prime, *F*(2,58) = 3.19, *p* = 0.05. Planned comparisons indicated that in the 250-ms SOA condition, the morphological prime statistically differed from the non-related prime [*t*(29) = 2.14, *p* = 0.04] and from the orthographic prime [*t*(29) = 2.42, *p* = 0.02]. The words preceded by morphological primes were recognized 86 ms faster than those preceded by non-related primes, and were recognized 109 ms faster than those preceded by orthographic primes. No further significant results were yielded for the other comparisons (all *p* > 0.10).

The ANOVA on fourth-grader data showed no main effect of SOA (*p* > 0.18). The Prime effect (*F*(2,62) = 6.7, *p* < 0.01) and the SOA × Prime interaction (*F*(2,62) = 3.22, *p* = 0.05) were significant though. Planned *t* tests showed that words preceded by morphological primes during the 250-ms SOA were recognized 110 ms faster than those preceded by non-related primes [*t*(31) = 3.25, *p* < 0.01] and were recognized 125 ms faster than those preceded by orthographic primes [*t*(31) = 3,9, *p* < 0,01]. No further significant results were yielded for the other comparisons (all *p* > 0.5).

For fifth graders, neither the main effect of SOA nor the SOA × Prime interaction were statistically significant in the ANOVA analysis (all *p* values > 0.52). The only statistically significant effect was that of Prime *F*(2,74) = 6.76, *p* < 0.01. When compared to words preceded by orthographic primes, words preceded by morphological primes were recognized 86 ms faster in the 250-ms SOA and 76 ms faster in the 60-ms SOA [*t*(37) = 3.69, *p* < 0.01 and *t*(37) = 2.42 *p* = 0.02, respectively]. When compared to words preceded by non-related primes, words preceded by morphological primes were recognized 76 ms faster in the 250-ms SOA [*t*(37) = 2.67, *p* = 0.01] and 62 ms faster in the 60-ms SOA (*p* = 0.059 being just significant).

Finally, in order to verify if there is any relation between morphological priming and morphological awareness, a correlation analysis was made between the effect of morphological priming and the scores in the Derived Word Analogy task. The effect of morphological priming was operationalized as the difference between the reaction time for words preceded by morphological primes and the reaction time for words preceded by orthographic primes or non-related primes, divided by the standard deviation of overall reaction time. For instance, if a participant responded to words preceded by morphological primes with reaction time “*X*” and responded to words preceded by orthographic primes with reaction time “*Y*,” the morphological priming effect for this participant would be the difference between *Y* and *X*, divided by the standard deviation of the participant overall reaction time in the LDT. Using this rationale, it was possible to calculate morphological priming effects in relation to non-related and orthographic primes by each type of SOA for each child. Table 2 presents the Pearson correlations between these data and the scores in the Derived Word Analogy task. There were no significant correlations between any morphological priming effect and morphological awareness scores in any school grade (all *p* values >0.09).

**Table 2:** Correlations between morphological priming scores and morphological awareness

Morphological awareness	SOA 60 ms			SOA 250 ms		
	NR-Morp	Ort-Morp	NR-Ort	NR-Morp	Ort-Morp	NR-Ort
2nd grade	−0.29	−0.28	0.00	−0.09	−0.20	0.11
3rd grade	−0.03	−0.18	0.18	−0.02	−0.28	0.28
4th grade	0.23	0.27	−0.02	0.22	0.10	0.15
5th grade	0.21	−0.07	0.28	−0.08	−0.02	−0.07

There were no significant correlations between morphological priming effects and morphological awareness (all *p* values >0.09)

*Abbreviations*: *NR-Morp* difference between the reaction time (RT) for morphological primes and the RT for non-related primes, *Ort-Morp* difference between the RT for orthographic primes and the RT for morphological primes, *NR-Ort* difference between the RT for non-related primes and the RT for orthographic primes

## Discussion

The central goal of the current study was to investigate the development of morphological priming in the reading of Brazilian Portuguese children. As observed in the ANOVA analysis, it is possible to find that morphological priming effects in Brazilian Portuguese have its onset as early as the second grade. After all, second graders recognized target words preceded by morphological primes faster than target words preceded by non-related primes in the 250-ms SOA. However, it is possible to argue that in this school grade, the morphological priming effect is very small, once it does not differ from the orthographic prime. Probably, morphological priming starts to develop in this school grade, but it is still poorly developed due to the child’s lack of experience with reading.

From third to fourth grade, in the 250-ms SOA condition, primes with morphological relation to the target words are recognized faster than non-related or orthographic primes. This can be taken as evidence that, in these school grades, the child’s reading experience is sufficient to decompose the words into its morphemic components during reading. This decomposing process is not limited to the extraction of the root properties of the words by simple letter sequencing, given that there is a significant difference between the orthographic and the morphological prime conditions. For example, the word “abriu” is facilitated by the prime “abriam” but not by the prime “abril”. Since both prime words (“abriam” e “abril”) share the same number of initial letters (four letters) with the target word (“abriu”), it is probably the morphological properties shared between “abriam” and “abriu” which boost the recognition of “abriu.” Thus, it seems that children from third and fourth grades are already able to process morphological units during visual word recognition.

The fifth grade seems to be the period of reading development in which the participants are sensible to morphological primes in both SOA conditions: 60 and 250 ms. In this school grade, when compared to words preceded by orthographic primes or by non-related primes, words preceded by morphological primes were recognized faster in the 250-ms SOA and in the 60-ms SOA. The fact that the *p* value (0.06) for the comparison between morphological and non-related primes in the 60-ms SOA was just close to the desired alpha level can be explained by lack of power considering that the sample size was not big.

The present results concerning morphological priming corroborate the view that there is some degree of morphological processing during visual word recognition (Domínguez et al. 2000). However, what is the nature of this morphological processing? Rastle and Davis (2008) proposed two distinct moments in visual word recognition: a morpho-orthographic moment, which would happen during the initial phase of perception (usually measured in studies with short SOAs), and a morpho-semantic moment, which would happen after that (usually measured in studies with longer SOAs). Assuming that Rastle and Davis are correct, we could argue that it is only in the fifth grade that morpho-orthographic decomposition has its onset in Brazilian Portuguese readers. After all, it was only in this school grade that morphological and orthographic primes statistically differed in the 60-ms SOA condition. Therefore, the morphological priming effects in the earlier grades were probably a result of morpho-semantic processing.

It makes sense to think of morpho-semantic processing developing earlier than morpho-orthographic processing because to benefit from morpho-orthographic units, the reader would need to have abstract orthographic patterns stored in his or her lexicon, and the abstraction of these units would depend on reading experience. As Rastle and Davis (2008) pointed out, morphological processing is likely related to the child’s experience with oral language, which permits the identification of repeated patterns that occur in the structures of words, as well as their experience with written language. A possible explanation for this developmental pattern is, once children already has some morphological knowledge derived from oral language, when they begin to read (by phonological recoding), they tend to “sound out” the words and to recover their meaning after it, and this would lead to a higher benefit from the semantic aspects of morphemes. As children acquire more reading experience, morphosyntatic patterns are abstracted and become automatic, boosting the processing of morphologically complex words.

It is important to notice that Casalis et al. (2009) results presented the same pattern of the current study considering the fourth graders, that is, fourth graders only presented morphological priming effects in the 250-ms SOA. Although Quémart et al. (2011) did find morphological priming effects in the 60-ms SOA in an experiment with third graders, it is important to notice they used different words in each prime condition, and this could confound word and priming effects. In the present study, lists were created and counterbalanced across experimental conditions so that in each list, a word was preceded by a type of prime and by a type of SOA. This procedure guarantees that each word works as its own control resulting in a better control of word effects. If the differences found between the present results and those of Quémart et al. were due to simple transcultural and linguistic factors, Casalis et al. would have found the morphological priming effect in their study’s short SOA condition, once it was also conducted in French-speaking children. Thus, it seems reasonable to conclude that morpho-orthographic priming probably occurs only after fourth grade.

In short, the present research suggests that morphological priming has its onset as early as the end of the second grade. In addition, the results suggest that morphological priming effects in the earlier grades are probably a result of morpho-semantic processing and that morpho-ortographic processing is a later acquisition, since only fifth graders were sensible to morphological primes in the 60-ms SOA condition. This is important evidence because as pointed out before by Chomsky and Halle (1968), it is reasonable to think that morphology would be important for reading in English because of its irregularities concerning grapheme-phoneme mapping. Thus, the present study shows that morphological processing plays a role in reading even in a more transparent language like Brazilian Portuguese.

An additional goal of the present study was to investigate if there is any correlation between morphological priming and morphological awareness in Brazilian Portuguese. No statistically significant correlation between the scores in the morphological awareness task (word analogy) and morphological priming effect during the LDT were observed in this study. More important, this lack of correlation holds for every school year investigated, that is from second to fifth grade. We do not have knowledge of other studies investigating the correlation between morphological awareness and morphological priming effects. Therefore, we cannot predict if these correlations should have been statistically significant. Thus, it seems to be a matter for further research.

## Conclusions

The present study is the first to investigate the development of morphological priming in Brazilian children in the beginning years of reading instruction. This is a very important cross-linguistic information, once other studies, which investigated morphological priming in children, were conducted in French. The present findings that morpho-semantic processing develops earlier than morpho-orthographic processing are important evidence for visual word recognition models to take into account. In addition, the fact that morphological processing has its onset as early as the end of the second grade highlights the importance of morphological processing for beginning readers and suggests pedagogical practices that foster morphological knowledge in children.

Finally, it is important to consider the limitations of the present study. The fact that the study used only one measurement of morphological awareness (derived word analogy task) might be considered a limitation as the hypothetical use of more measures might have made it possible to calculate a composed and more accurate measure of this variable. In any case, it is also relevant to point out that the Derived Word Analogy task used in this study did not present any outliers and had normal distribution. Another limitation of this study is the sample restricted to students from private schools. Thus, an important issue for future research would be to evaluate if public school students present the same developmental pattern for the morphological priming effect.

## Additional file

Additional file 1: Lists of lexical decision task stimuli. (DOCX 55 kb)
