# Supplementary material for: Morphological priming development in Brazilian Portuguese-speaking children
Source: Psicol Reflex Crit. 2017 Feb 20;30:4. doi: 10.1186/s41155-017-0058-8 (PMC6967254; doi:10.1186/s41155-017-0058-8)
Supplement: Supplementary file 1 — Lists of lexical decision task stimuli. (DOCX 55 kb) [file 41155_2017_58_MOESM1_ESM.docx]

LISTS OF LEXICAL DECISION TASK STIMULI

**Second grade**

| **TARGET** | **MORPHOLOGICAL PRIME** | **ORTOGRAFIC PRIME** | **NON-RELATED PRIME** | **PSEUDOWORDS PRIME** | **PSEUDOWORDS** |
| --- | --- | --- | --- | --- | --- |
|  |  |  |  |  |  |
| ALTA | altura | altar | maço | afeto | CAPARA |
| AMOR | amores | amora | sino | ameixa | PONDE |
| ANDAR | andou | andorinha | raça | ampola | PETRI |
| ARME | armar | armazém | gozado | aposta | AMACA |
| BELA | beleza | belém | regime | atleta | LISCO |
| BICO | bicada | bicho | filtro | azeite | INONE |
| BOLO | bolinhos | bolada | pista | basquete | POTU |
| CAMA | caminha | camarão | concha | besta | ROMES |
| CEDO | cedinho | cedilha | mente | bexiga | TADE |
| COLA | colou | colabora | surdo | bigode | MORU |
| COPO | copinho | copiou | risco | bloco | ATAFA |
| DEDO | dedal | dedicação | tonto | boba | LETIM |
| DIGA | disse | digital | turno | bodoque | RAMIR |
| FACA | faqueiro | face | espanto | bote | TULO |
| FILA | fileira | filha | tombo | brejo | PLOCA |
| FOFA | fofura | fofoca | balde | buzina | FRINO |
| LATE | latido | lateral | ervilha | cabelo | GOTO |
| LEVA | levaram | levantam | salto | camelo | BIBE |
| MAÇA | maciera | macio | lenço | cartela | PROUCA |
| NOVA | novidade | novela | bengala | cebola | NEMA |
| PEDE | pediam | pedestre | salsa | cego | REBA |
| PESO | pesado | pesadelo | brava | cela | SERRU |
| PULA | pulei | pulga | campina | celeste | CRIBO |
| ROLA | roleta | rolha | prata | cerveja | ELEME |
| SACO | sacola | sacode | destino | choque | CADO |
| SAIR | saída | saiba | grade | colete | LONAR |
| TREM | trenzinho | tremer | grilo | cova | DRADO |
| ABRIU | abriam | abril | trave | danada | CETU |
| ACHAR | achou | achatado | trigo | delicada | FONA |
| AVISO | avisou | avistou | traje | ervilha | VODA |
| BEBER | bebeu | bebê | dupla | esquerdo | NICER |
| CAMPO | campina | campainha | ladra | favela | MANE |
| CANTA | cantora | cantil | magra | febre | BISCO |
| CAPAZ | capacidade | capacete | ciclo | forca | LAFÕES |
| CARNE | carnívoro | carneiro | viúva | forno | UREVA |
| CHEIA | cheios | cheira | fiapo | gabinete | TARE |
| CLARO | clarear | clarim | fiado | gaveta | TOZI |
| COMEU | comer | comecei | viúva | gorjeta | TARIU |
| CUBRA | coberta | cubo | monarca | honesto | ZEIO |
| DENTE | dentista | dentro | feminina | leque | UDÃOS |
| LAVAR | lavou | lavoura | antiga | limonada | QUIVU |
| LIVRE | livrar | livro | estima | martelo | VINEL |
| MEDIR | medido | médico | espiga | miolo | PROCA |
| PASSA | passei | pássaro | ensino | moderna | MEPO |
| PATA | patada | paterna | redonda | mosquito | RILOU |
| SABÃO | ensaboar | sabia | ditongo | negra | COGER |
| SABIA | sabem | sabor | recurso | peteca | MAICA |
| SENTE | sentido | sentença | cimento | pipoca | DONHO |
| SONHO | sonhava | risonho | humilde | polvo | FIBEDA |
| SUSTO | assusta | sustenta | nublado | poste | JOTELO |
| UNIÃO | unem | unha | veludo | preguiça | UJADO |
| VALOR | valem | valente | tucano | repleto | PUMPE |
| VENDO | revendo | vendado | relato | sabida | NAMA |
| COLHEU | colheita | colher | retalho | sapeca | OUBRE |
| CONTOU | reconta | continua | caçada | sarjeta | LOFRA |
| FORMAS | formou | formiga | mochila | tijolo | BENHO |
| MORAVA | moradia | morango | caçula | tombo | LEBRAL |
| PEDAÇO | pedacinho | pedal | cigana | trena | VARI |
| CORRIDA | correu | correio | gemada | universo | TUZÃO |
| PADEIRO | padaria | padre | cilada | zebu | CADIA |

**Third grade**

| **TARGET** | **MORPHOLOGICAL PRIME** | **ORTOGRAFIC PRIME** | **NON-RELATED PRIME** | **PSEUDOWORDS PRIME** | **PSEUDOWORDS** |
| --- | --- | --- | --- | --- | --- |
| ABRIU | abriam | abril | maço | afeto | CAPARA |
| ACHAR | achou | achatada | sino | ameixa | PONDE |
| ALTA | altura | altar | raça | ampola | PETRI |
| AMOR | amores | amora | gozado | aposta | AMACA |
| ANDAR | andou | andorinha | regime | atleta | LISCO |
| ARME | armar | armazém | filtro | azeite | INONE |
| BEBER | bebeu | bebê | pista | basquete | POTU |
| BELEZA | belas | belém | concha | besta | ROMES |
| BICO | bicada | bicho | mente | bexiga | TADE |
| BOLO | bolinho | bolona | surdo | bigode | MORU |
| CAMA | caminha | camarão | risco | bloco | ATAFA |
| CANTE | cantora | cantil | tonto | boba | LETIM |
| CAPAZ | capacidade | capacete | turno | bodoque | RAMIR |
| CARNE | carnívoro | carneiro | espanto | bote | TULO |
| CEDO | cedinho | cedilha | tombo | brejo | PLOCA |
| CHEIA | cheios | cheira | balde | buzina | FRINO |
| CLARO | clarear | clarim | ervilha | cabelo | GOTO |
| COLE | colou | colabora | salto | camelo | BIBE |
| COLHEU | colheita | colher | lenço | cartela | PROUCA |
| COMIDA | comer | comecei | bengala | cebola | NEMA |
| CONTOU | reconte | continua | salsa | cego | REBA |
| CORREU | corrida | correio | brava | cela | SERRU |
| DEDO | dedal | dedicação | campina | celeste | CRIBO |
| FORMAS | forme | formiga | prata | cerveja | ELEME |
| LEVAR | levaram | levantam | grade | colete | LONAR |
| LIVRE | livrar | livro | grilo | cova | DRADO |
| MEDIR | medido | médica | trave | danada | CETU |
| MORAVA | moradia | morango | trigo | delicada | FONA |
| NOVOS | novidade | novela | traje | ervilha | VODA |
| PASSAVA | passei | pássaro | dupla | esquerdo | NICER |
| PATA | patinha | paterna | ladra | favela | MANE |
| PEDAÇO | pedacinho | pedal | magra | febre | BISCO |
| PEDIU | pedem | pedestre | ciclo | forca | LAFÕES |
| PESO | pesado | pesadelo | viúva | forno | UREVA |
| PULA | pulei | pulga | fiapo | gabinete | TARE |
| SENTIDO | sente | sentença | fiado | gaveta | TOZI |
| VALE | valor | valente | viúva | gorjeta | TARIU |
| VENDO | revendo | vendados | monarca | honesto | ZEIO |
| BANHO | banhava | rebanho | feminina | leque | UDÃOS |
| BRANCO | branquinho | brando | revista | limite | LUSAM |
| CARROS | carreta | carrega | antiga | limonada | QUIVU |
| CASINHA | casa | casaco | estima | martelo | VINEL |
| CHAMADA | chamou | chaminé | espiga | miolo | PROCA |
| CIRCULE | círculo | circo | ensino | moderna | MEPO |
| COLEÇÃO | coleciona | colega | redonda | mosquito | RILOU |
| COMPRA | comprei | comprida | ditongo | negra | COGER |
| CÓPIA | copiou | copinhos | recurso | peteca | MAICA |
| CORDA | cordão | cordeiro | cimento | pipoca | DONHO |
| CURIOSO | curiosas | curió | humilde | polvo | FIBEDA |
| DEVEM | dívida | devagar | nublado | poste | JOTELO |
| ENTRAR | entrou | entrega | veludo | preguiça | UJADO |
| ESQUEÇA | esqueceu | esqueleto | tucano | repleto | PUMPE |
| FEIO | feiúra | feitiço | relato | sabida | NAMA |
| FOGO | fogão | foge | retalho | sapeca | OUBRE |
| FOME | faminto | fomos | caçada | sarjeta | LOFRA |
| GIRA | girando | girafa | mochila | tijolo | BENHO |
| MACACO | macaquisse | macarrão | caçula | tombo | LEBRAL |
| OLHOU | olhando | piolho | cigana | trena | VARI |
| PENSA | pensou | despensa | gemada | universo | TUZÃO |
| SOBROU | sobrar | sobremesa | cilada | zebu | CADIA |

**Fourth grade**

| **TARGET** | **MORPHOLOGICAL PRIME** | **ORTOGRAFIC PRIME** | **NON-RELATED PRIME** | | **PSEUDOWORDS PRIME** | **PSEUDOWORDS** |
| --- | --- | --- | --- | --- | --- | --- |
| ABRIU | Abriam | abril | maço | | afeto | CAPARA |
| ACHAR | Achou | achatado | sino | | ameixa | PONDE |
| ALTA | Altura | altar | raça | | ampola | PETRI |
| ALTERA | Alterado | alternam | gozado | | aposta | AMACA |
| AMOR | Amores | amora | regime | | atleta | LISCO |
| ANDAR | Andou | andorinha | filtro | | azeite | INONE |
| ARME | Armar | armazém | pista | | basquete | POTU |
| AZUIS | Azulado | azulejo | concha | | besta | ROMES |
| BARRA | Barrinha | barraco | mente | | bexiga | TADE |
| BATEU | Batida | batata | surdo | | bigode | MORU |
| BEBER | Bebeu | bebê | risco | | bloco | ATAFA |
| BELA | beleza | belém | tonto | | boba | LETIM |
| BRANCO | Branquinho | brando | turno | | bodoque | RAMIR |
| CAMA | Caminha | camarão | espanto | | bote | TULO |
| CAMPOS | Campina | campainha | tombo | | brejo | PLOCA |
| CANTAR | Cantora | cantil | balde | | buzina | FRINO |
| CARROS | Carreta | carrega | ervilha | | cabelo | GOTO |
| CASINHA | Casa | casaco | salto | | camelo | BIBE |
| CEDO | Cedinho | cedilha | lenço | | cartela | PROUCA |
| CHAMADA | Chamou | chaminé | bengala | | cebola | NEMA |
| CHEIA | Cheios | cheira | salsa | | cego | REBA |
| CLARO | Clareza | clarim | brava | | cela | SERRU |
| COLE | Colou | colabora | campina | | celeste | CRIBO |
| COLEÇÃO | Coleciona | colega | prata | | cerveja | ELEME |
| COMI | Comer | comecei | destino | | choque | CADO |
| COMPRA | Compre | comprida | grade | | colete | LONAR |
| CONVITE | Convidada | convicto | trave | | danada | CETU |
| COPO | Copinho | copiou | trigo | | delicada | FONA |
| CORREU | Corrida | correio | traje | | ervilha | VODA |
| DEVEMOS | Dívida | devagar | dupla | | esquerdo | NICER |
| ENTRAR | Entrou | entrega | ladra | | favela | MANE |
| ESCOLAS | Escolar | escoltar | magra | | febre | BISCO |
| ESCRITA | Escreva | escravos | ciclo | | forca | LAFÕES |
| ESQUEÇA | Esqueceu | esqueleto | viúva | | forno | UREVA |
| FEIO | Feiúra | feitiço | fiapo | | gabinete | TARE |
| FOGO | Fogão | foge | fiado | | gaveta | TOZI |
| FOME | Faminto | fomos | viúva | | gorjeta | TARIU |
| FORMAS | Formou | formiga | monarca | | honesto | ZEIO |
| GIRA | Girando | girafa | feminina | | leque | UDÃOS |
| INDICAR | indique | índio | revista | | limite | LUSAM |
| JEITO | Ajeita | sujeito | antiga | | limonada | QUIVU |
| LEVA | Levaram | levantam | estima | | martelo | VINEL |
| LIVRE | livrar | livro | | espiga | miolo | PROCA |
| MAÇA | maciera | macio | | ensino | moderna | MEPO |
| MEDIR | medido | médico | | redonda | mosquito | RILOU |
| MORAVA | moradia | morango | | ditongo | negra | COGER |
| NOVAS | novidade | novela | | recurso | peteca | MAICA |
| OLHOU | olhando | piolho | | cimento | pipoca | DONHO |
| PATA | patinha | paterna | | humilde | polvo | FIBEDA |
| PEDAÇOS | pedacinho | pedalando | | nublado | poste | JOTELO |
| PEDE | pediam | pedestre | | veludo | preguiça | UJADO |
| PENSAR | Pensou | despensa | | tucano | repleto | PUMPE |
| SABIA | Sabem | sabor | | relato | sabida | NAMA |
| SAIR | Saída | saiba | | retalho | sapeca | OUBRE |
| SENTIU | Sentidos | sentença | | caçada | sarjeta | LOFRA |
| SONHO | Sonhava | risonho | | mochila | tijolo | BENHO |
| SUSTO | Assusta | sustenta | | caçuça | tombo | LEBRAL |
| TREM | Trenzinho | tremer | | cigana | trena | VARI |
| UNIÃO | Unem | unha | | gemada | universo | TUZÃO |
| VENDO | Revender | chovendo | | cilada | zebu | CADIA |

**Fifth grade**

| **TARGET** | **MORPHOLOGICAL PRIME** | **ORTOGRAFIC PRIME** | **NON-RELATED PRIME** | **PSEUDOWORDS PRIME** | **PSEUDOWORDS** |
| --- | --- | --- | --- | --- | --- |
| ABRIU | Abriam | abril | maço | afeto | CAPARA |
| ACHAR | Achou | achatado | sino | ameixa | PONDE |
| ALTA | Altura | altar | raça | ampola | PETRI |
| AMOR | Amores | amora | gozado | aposta | AMACA |
| AZUIS | Azulado | azulejo | regime | atleta | LISCO |
| BARRA | Barrinha | barraco | filtro | azeite | INONE |
| BELEZA | Belas | belém | pista | basquete | POTU |
| BOLO | Bolinho | bola | concha | besta | ROMES |
| BRANCO | Branquela | brando | mente | bexiga | TADE |
| CAMA | Caminha | camarão | surdo | bigode | MORU |
| CAMPOS | Campina | campainha | risco | bloco | ATAFA |
| CANTAR | Cantora | cantil | tonto | boba | LETIM |
| CAPAZ | Capacidade | capado | turno | bodoque | RAMIR |
| CARNE | Carnívoro | carneiro | espanto | bote | TULO |
| CEDO | Cedinho | cedilha | tombo | brejo | PLOCA |
| CHAMADA | Chamou | chaminé | balde | buzina | FRINO |
| CHEIA | Cheios | cheira | ervilha | cabelo | GOTO |
| CIRCULE | Círculos | circo | salto | camelo | BIBE |
| CLARO | Clareza | clarim | lenço | cartela | PROUCA |
| COMER | Comi | comecei | bengala | cebola | NEMA |
| CORDA | Cordão | cordeiro | brava | cela | SERRU |
| CORREU | corrida | correio | campina | celeste | CRIBO |
| DENTES | dentista | dentro | prata | cerveja | ELEME |
| DEVEMOS | dívida | devagar | destino | choque | CADO |
| DIZIA | disse | dizimou | grade | colete | LONAR |
| ENTRAR | entrou | entrega | grilo | cova | DRADO |
| ESCOLHA | escolhe | escolar | trave | danada | CETU |
| ESCRITA | escrevam | escravos | trigo | delicada | FONA |
| FOGO | fogão | foge | traje | ervilha | VODA |
| FOME | faminto | fomos | dupla | esquerdo | NICER |
| FORMAS | formou | formol | ladra | favela | MANE |
| INDICAR | indique | índio | magra | febre | BISCO |
| LEVAR | levou | levantam | viúva | forno | UREVA |
| LIVRE | livrar | livro | fiapo | gabinete | TARE |
| MACACO | macaquisse | macarrão | fiado | gaveta | TOZI |
| MEDIR | medido | médico | viúva | gorjeta | TARIU |
| MORAVA | moradia | morango | monarca | honesto | ZEIO |
| NOVAS | novidade | novela | feminina | leque | UDÃOS |
| OLHOU | olhando | piolho | revista | limite | LUSAM |
| PASSAR | passei | pássaro | antiga | limonada | QUIVU |
| PEDAÇOS | pedacinho | pedalando | estima | martelo | VINEL |
| PEDE | pediam | pedestal | espiga | miolo | PROCA |
| PENSA | pensou | despensa | ensino | moderna | MEPO |
| PESO | pesado | pesadelo | redonda | mosquito | RILOU |
| SABENDO | sabiam | sabor | ditongo | negra | COGER |
| SAIR | saída | saiba | recurso | peteca | MAICA |
| SENTIDO | sente | sentença | cimento | pipoca | DONHO |
| VALE | valor | valente | humilde | polvo | FIBEDA |
| VENDO | revender | chovendo | nublado | poste | JOTELO |
| VOLTAR | voltou | revolta | veludo | preguiça | UJADO |
| TORNOU | tornava | torneio | tucano | repleto | PUMPE |
| TOMAR | tomado | tomate | relato | sabida | NAMA |
| SAPATO | sapateiro | sapa | retalho | sapeca | OUBRE |
| PROVA | prove | provoca | caçada | sarjeta | LOFRA |
| PORTA | portão | porto | mochila | tijolo | BENHO |
| LEITURA | leia | leite | caçuça | tombo | LEBRAL |
| GRAMA | gramado | gramática | cigana | trena | VARI |
| DESTAQUE | destacado | destas | gemada | universo | TUZÃO |
| CENTO | centena | central | cilada | zebu | CADIA |
| PARAR | parou | parecia | secreto | seda | BROGA |
